# Effect of Gelatin Coating Enriched with Antioxidant Tomato By-Products on the Quality of Pork Meat

**DOI:** 10.3390/polym12051032

**Published:** 2020-05-02

**Authors:** Marta Gallego, Milagros Arnal, Pau Talens, Fidel Toldrá, Leticia Mora

**Affiliations:** 1 Instituto de Agroquímica y Tecnología de Alimentos (CSIC), Avenue Agustín Escardino 7, 46980 Paterna (Valencia), Spain; margalib@upv.es (M.G.); miarsa@upv.es (M.A.); ftoldra@iata.csic.es (F.T.); 2 Departamento de Tecnología de Alimentos, Universitat Politècnica de València, Camino de Vera s/n, 46022 Valencia, Spain; pautalens@tal.upv.es

**Keywords:** meat, coating, by-products, peptides, antioxidant activity, lipid oxidation, cooking

## Abstract

The use of edible biopolymers and natural additives obtained from food processing by-products is a sustainable strategy for food packaging applications. Gelatin is a biopolymer with great potential as a coating due to its low cost, high availability, and technological and functional properties. Among them, gelatin can be used as a carrier of bioactive compounds such as antioxidants, which can retard oxidation processes and thus extend the shelf-life of highly-perishable products. This study evaluated the effect of gelatin coating enriched with antioxidant tomato by-products hydrolysate (TBPH) on the quality of pork meat during cold storage. Results showed that TBPH obtained from Alcalase hydrolysis presented antioxidant activity with good stability against cooking. Additionally, chromatographic and mass spectrometry techniques, as well as *in silico* analysis, were used for the peptidomic characterisation of TBPH. The application of enriched gelatin coating on meat led to some physicochemical changes including increased weight loss and colour differences; however, the pH and water activity, which control meat spoilage, were maintained during storage. Moreover, coating prevented lipid oxidation of meat, and enriched-coated meat presented high antioxidant activity after cooking. These results suggest the positive role of gelatin coating enriched with TBPH in extending the shelf-life of meat during storage.

## 1. Introduction

Fresh meat is a highly-perishable product due to its biological composition, and its shelf-life is dependent on pre-slaughter, processing, and post-processing conditions. The first indicators of meat deterioration for consumers are appearance and aroma changes, mainly resulting from water exudates, colour loss due to myoglobin oxidation, rancidity caused by lipid oxidation, and microbial spoilage [1]. 

The application of films and coatings in the food packaging industry has noticeably increased in the last few decades to maintain food safety and quality and thus extend its shelf-life. The consumer demand for preserving food in a natural way has led to extend the use of edible biopolymers obtained from industrial by-products. In this regard, gelatin is being widely employed in food packaging because of its low cost, high availability, sensory acceptability, and functional and technological properties. Moreover, it results advantageously in terms of environmental sustainability, as gelatin is obtained from the partial hydrolysis of collagen protein mainly present in animal by-products such as skin, bones, tendons, and connective tissues [2,3]. The main characteristics of gelatin are its high content of glycine, proline, and hydroxyproline amino acids and the mixture of single- and double-unfolded chains with hydrophilic character. During gelation, the chains undergo a conformational de-folding transition and tend to recover the triple-helix structure of collagen [4]. Gelatin coating has been used to extend the quality and shelf-life of meat products due to its action as a barrier to water and oxygen, thereby reducing purge, colour loss, and aroma deterioration [5,6,7]. Furthermore, the coating can be an excellent carrier for incorporating an extensive variety of compounds such as crosslinkers, strengthening agents, plasticisers, nutrients, antioxidants, antimicrobials, colourants, and flavours, which are mainly used to improve the functional properties of coatings and the shelf-life of food products [2,3]. The addition of natural antioxidant compounds in gelatin-based films has shown to be effective for the prolongation of food shelf-life based on retarding oxidation processes affecting proteins, pigments, and lipids. In this regard, phenolic compounds, essential oils, α-tocopherol, polysaccharides, and peptides have been widely employed to enrich gelatin films [7,8,9,10,11].

Tomato processing industries generate a considerable amount of protein-rich by-products, mainly skins and seeds, which could be used as a potential source of bioactive peptides. In this regard, tomato waste hydrolysates produced by fermentation with *Bacillus subtilis* have been reported to exert antibacterial, antioxidant, and ACE inhibitory activities [12,13]. Also, the hydrolysis of tomato seeds using Alcalase enzyme showed great antioxidant activity [14]. Considering this background, hydrolysates from tomato by-products could be added to gelatin coating in order to improve its antioxidant properties, giving an added-value to these by-products. 

The use of edible coatings and antioxidant agents obtained from by-products would be a natural and sustainable strategy to improve the quality of food products. This provides an added value to by-products, giving a new application that increases economic value, decreasing the environmental impact and helping the transition toward a more sustainable bioeconomy in the food industry. Thus, the present study aims to evaluate the effect of a gelatin coating enriched with tomato by-products hydrolysate on the quality of fresh pork meat during cold storage. The effect of cooking on the antioxidant activity of tomato by-products hydrolysate was evaluated, and chromatography, mass spectrometry, and bioinformatic tools were employed for peptidomic characterisation. Then, several physicochemical parameters of gelatin-coated meats, the effect of coating on lipid oxidation of meat during storage, as well as the effect of cooking on antioxidant activity of coated meat samples were assessed.

## 2. Materials and Methods

### 2.1. Chemicals and Reagents

Protease enzyme from *Bacillus licheniformis* (Alcalase^®^ ≥ 2.4 U/g) and gelatin from porcine skin were purchased from Sigma-Aldrich, Co. (St. Louis, MO, USA). Chemicals for antioxidant activity: 2,2-diphenyl-1-picrylhydrazyl (DPPH), potassium ferricyanide, ferric chloride, and 2,2′-azino-bis(3-ethylbenzothiazoline-6-sulfonic acid) diammonium salt (ABTS) were purchased from Sigma-Aldrich, Co. (St. Louis, MO, USA), whereas potassium persulfate and butylated hydroxytoluene (BHT) were from Panreac Quimica S.A.U. (Barcelona, Spain). Chemicals for lipid oxidation: 2-thiobarbituric acid (TBA), 1,1,3,3-tetramethoxypropan (TMP), and trichloroacetic acid (TCA) were from Sigma-Aldrich, Co. (St. Louis, MO, USA). All other reagents and chemicals were of analytical grade.

### 2.2. Preparation of the Tomato By-Products Hydrolysate

Tomatoes of long-life variety obtained from a local market were blanched at 80 °C for 1 min in a water-bath in order to easily obtain skins and seeds, which were freeze-dried and grounded to obtain a powder.

#### 2.2.1. Optimisation of Enzymatic Hydrolysis

A total of 0.3 g of tomato by-products powder was mixed with 10 mL of Tris-HCl buffer (50 mM, pH 8) and subjected to enzymatic hydrolysis by adding 100 μL of Alcalase enzyme. The hydrolysis was carried out at 50 °C and pH 8 in a digestor (Carousel 6 Plus Reaction Station, Radleys, Saffron Walden, UK). Different hydrolysis times were tested: 30 min, 60 min, 120 min, 240 min, 24 h, and 48 h. In order to stop the enzymatic reaction, samples were heated in a water bath for 2 min at 95 °C. Then, precipitation of proteins was done by adding 3 volumes of ethanol and maintaining the samples at 4 °C for 20 h. After centrifugation at 10,000 rpm and 4 °C for 10 min, the supernatant was dried in a rotatory evaporator, lyophilised, and stored at −20 °C until used. This lyophilised powder is referred to as TBPH (tomato by-products hydrolysate) and assayed for antioxidant activity at 10 mg/mL in order to confirm the best hydrolysis conditions.

#### 2.2.2. Cooking of TBPH

The stability of the antioxidant activity of the TBPH against cooking was evaluated at different sample concentrations (1, 5, and 10 mg/mL). The TBPH was cooked in a water bath at 70 °C for 5, 10, and 15 min, and the antioxidant activity was evaluated in order to optimise the conditions to achieve the highest activity.

#### 2.2.3. Peptidomic Characterisation

##### Size-Exclusion Chromatography (SEC)

The TBPH showing the highest antioxidant activity was fractionated by size-exclusion chromatography (SEC). Thus, 5 mL of TBPH (25 mg/mL) was filtered through a 0.45 μm nylon membrane filter and injected in a Sephadex G25 gel filtration column (2.5 × 65 cm, Amersham Biosciences, Uppsala, Sweden), using 0.01 N HCl as eluent at a flow rate of 15 mL/h and 4 °C. Fractions of 5 mL were automatically collected and monitored at 214, 254, and 280 nm (UV-visible spectrophotometer 8453, Agilent Technologies, Palo Alto, CA, USA). Lastly, fractions from 26 to 130 were grouped into three (final elution volume of 15 mL) and assayed for antioxidant activity.

##### Reversed-Phase High Performance Liquid Chromatography (RP-HPLC)

The TBPH fractions obtained from SEC exhibiting remarkable antioxidant activity were pooled together, lyophilised, and purified by reversed-phase high-performance liquid chromatography (RP-HPLC). For that, sample was resuspended in 280 μL of bidistilled water and 100 μL was injected into an Agilent 1100 HPLC system (Agilent Technologies, Palo Alto, CA, USA). The column was a Symmetry C18 (5 μm, 4.6 × 250 mm; Waters, Milford, MA, USA), and mobile phases were 0.1% trifluoroacetic acid (TFA) as solvent A and 0.085% TFA in acetonitrile:water (60:40, *v/v*) as solvent B. The gradient used for peptide elution was 100% A for 2 min, 0–5% B for 8 min, 5–80% B for 5 min, and 80–100% B for 10 min, at a flow rate of 1 mL/min. The separation was monitored using a diode array detector at 214 nm and 280 nm. Fractions of 1 mL were collected and assayed for antioxidant activity. Those fractions showing remarkable activity were freeze-dried for further tandem mass spectrometry analysis.

##### Peptide Identification by Tandem Mass Spectrometry

The identification of the peptides was done by nanoliquid chromatography-tandem mass spectrometry (nLC-MS/MS) using an Eksigent Nano-LC Ultra 1D Plus system (Eksigent of AB Sciex, Redwood city, CA, USA) coupled to the quadrupole/time-of-flight (Q-ToF) TripleTOF^®^ 5600+ system (AB Sciex Instruments, Framingham, MA, USA) with a nanoelectrospray ionisation source (ESI). Lyophilised samples were resuspended to reach a concentration of 10 mg/mL and 2 μL were injected into the nLC-MS/MS system. Firstly, samples were pre-concentrated on an Eksigent C18 trap column (3 μm, 350 × 500 μm; Eksigent of AB Sciex, Redwood city, CA, USA) at a flow rate of 3 μL/min for 5 min using 0.1% TFA as mobile phase, and then eluted on a nano-HPLC capillary column (3 μm, 75 μm × 12.3 cm, C18; Nikkyo Technos Co, Ltd., Tokyo, Japan). Mobile phases were 0.1% formic acid (FA) as solvent A and acetonitrile as solvent B. A linear gradient from 5 to 35% B for 90 min was used for peptide elution at a flow rate of 0.30 μL/min. Samples were analysed in the nanoESI-Q-ToF system with the following operating conditions: ESI voltage 2.8 kV, positive polarity, data-dependent acquisition mode, survey MS1 scans from 350–1250 m/z for 250 ms, MS2 experiments for 100–1500 m/z for 50 ms in ‘high sensitivity’ mode, and 1–5 charged ions. Data analysis was performed using Mascot Distiller v2.7 software (Matrix Science, Inc., Boston, MA, USA; http://www.matrixscience.com). The database search was done through Mascot Distiller v2.7.1 software in the Uniprot protein database (http://www.uniprot.org) selecting Green plant taxonomy, no enzyme specificity, and mass tolerance of 50 ppm in MS mode and 0.3 Da for MS/MS ions. 

##### *In Silico* Analysis

The BIOPEP database (http://www.uwm.edu.pl/biochemia/index.php/en/biopep) was used in the search of similar sequences previously identified as antioxidant peptides [15].

The potential bioactivity of the peptides was predicted using the Peptide Ranker software (http://distilldeep.ucd.ie/PeptideRanker/), which scored peptides from 0 to 1 so that higher value means a higher probability of being bioactive. The prediction is based on the fact that bioactive peptides have specific structural characteristics and amino acid sequences that endow their particular activities [16].

Peptide toxicity and physicochemical properties such as hydrophobicity, hydrophilicity, steric hindrance, and amphipathicity were evaluated using the ToxinPred software (http://crdd.osdd.net/raghava/toxinpred/). Peptide toxicity was predicted mainly according to the amino acid composition and position of peptides [17]. 

The potential peptide allergenicity was predicted using the AllerTOP v. 2.0 software (http://www.ddg-pharmfac.net/AllerTOP/index.html), which classified peptides as probable allergen or non-allergen based on their physicochemical properties [18].

### 2.3. Preparation of Pork Loin Samples Coated with Gelatin and Gelatin Enriched with TBPH

#### 2.3.1. Gelatin Solution Preparation and Enrichment with TBPH

The gelatin was dissolved in bidistilled water (8%; *w/v*) at approximately 40 °C with continuous stirring for 30 min to allow for the gelatin powder to dissolve into solution. Part of the prepared gelatin solution was enriched with the lyophilised TBPH, which was added to reach a final concentration of 10 mg/mL.

#### 2.3.2. Gelatin Coating of Pork Loin

Fresh pork loin steaks, muscle *Longissimus dorsi*, were prepared by removing the external fat and connective tissue. Meat samples were cut to obtain cubes (2 × 2 × 2 cm) that were used for controls (uncoated), coated with the gelatin solution, and coated with the gelatin enriched with TBPH. 

For coated samples, the gelatin solution was tempered to 28–30 °C and meat cubes were immersed in the solution twice (one time for 10 s and next time for 5 s) in order to get an efficient coating. After the application of the gelatin, the samples were allowed to sit for approximately 20 min to allow the gelatin to cool and set. The same procedure was carried out by coating meat samples with the gelatin solution enriched with TBPH.

Uncoated and coated meat samples were stored at 4 °C for 13 days, taking samples at different times during storage (0, 3, 6, 8, and 13 days) in order to measure several physicochemical parameters and lipid oxidation. In addition, the stability against cooking of the antioxidant activity of uncoated and coated meat samples was evaluated at times 0, 6, and 10 days, in duplicate. For this purpose, 0.5 g of the sample was cooked at 70 °C for 5 min in a water bath. Then, the sample was deproteinised by adding 3 volumes of ethanol and keeping at 4 °C for 20 h, centrifuged at 10,000 rpm and 4 °C for 10 min, and the supernatant was dried in a rotatory evaporator and freeze-dried. The obtained lyophilisate was resuspended in 1 mL of water, and the antioxidant activity was assessed.

### 2.4. Antioxidant Activity

The antioxidant activity for TBPH was evaluated by DPPH radical scavenging activity and ferric-reducing antioxidant power (FRAP). On the other hand, meat samples including uncoated, gelatin-coated, and enriched gelatin-coated, were assayed by FRAP and ABTS radical scavenging capacity instead of DPPH radical scavenging activity due to the high hydrophilicity of meat extracts, which resulted in important interferences using the DPPH method.

#### 2.4.1. DPPH Radical Scavenging Activity

The DPPH activity was determined according to the method described by Bersuder et al. [19]. Thus, 100 μL of each sample, 500 μL of ethanol and 125 μL of DPPH solution (0.02% of DPPH in ethanol) were mixed and incubated in the dark for 60 min. The reduction of DPPH radicals was measured at 517 nm, and BHT was used as a positive control. The antioxidant activity was expressed as the percentage of DPPH radical scavenging activity.

#### 2.4.2. Ferric-reducing Antioxidant Power (FRAP)

The ferric-reducing power was evaluated based on the ability to reduce ferric iron (Fe^3+^) to ferrous iron (Fe^2+^) [20]. For that, 70 μL of each sample, 70 μL of phosphate buffer (200 mM, pH 6.6) and 70 μL of potassium ferricyanide (10 mg/mL) were mixed and was incubated at 50 °C for 20 min. Then, 70 μL of TCA (100 mg/mL) was added, and the mixture was centrifuged at 4000 rpm and 4 °C for 10 min. Subsequently, 140 μL of the supernatant was mixed with 28 μL of ferric chloride (1 mg/mL) and 140 μL of bidistilled water, and the absorbance was measured at 690 nm. BHT was used as a positive control. Higher absorbance values indicate higher antioxidant activity. 

#### 2.4.3. ABTS Radical Scavenging Capacity

The ABTS radical scavenging capacity was evaluated as described by Re et al. [21] with some modifications. Briefly, 7 mM of ABTS dissolved in 2.45 mM of potassium persulfate was kept in the dark at room temperature for 12–16 h to produce ABTS+. The ABTS+ solution was diluted with 50 mM of phosphate buffer saline (PBS, pH 7.4) until it reached an absorbance of 0.70 ± 0.02 at 734 nm. Then, 10 μL of sample in water (1:1, *v/v*) was mixed with 990 μL of ABTS+ solution, and the absorbance was measured at 734 nm after incubation for 6 min. Ascorbic acid was used as positive control and PBS as a negative control. The antioxidant activity was expressed as percentage inhibition of ABTS radical scavenging activity.

### 2.5. Weight Loss, pH, Water Activity and Colour Measurements

The weight loss, pH, water activity and colour were evaluated (n = 6) in the uncoated (control) and TBPH-enriched gelatin-coated samples at different days during storage (0, 3, 6, 8, and 13 days). In order to compare samples in the same conditions, the gelatin was removed in coated samples immediately before performing the measurements.

The weight loss was calculated by Equation (1), where W_0_ is the initial weight of the sample (day 0) and Ws is the weight of the sample at a certain storage day.

Weight loss (%) = [(W_0_ − Ws)/W_0_] × 100 (1)

The pH was measured using a pH-meter (920 Expandable Ion Analyzer, Orion Research, Inc., Boston, MA, USA) in 1 g of each sample homogenised with 20 mL of bidistilled water, at room temperature.

The water activity (a_w_) was determined at 25 °C using an Aqua Lab series 3 water activity meter (Decagon Devices, Inc., Pullman, WA, USA), previously calibrated with saturated saline solutions.

The colour was determined using a Konica Minolta CM-2600d spectrophotometer (Konica Minolta Sensing, Inc., Osaka, Japan). An average value of colour for each sample was determined by taking observations from the four sides of the meat cube. Samples were placed on a white standard plate and colour coordinates L*, a* and b* in the CIELab space by using the standard light source D65 and standard observer 10° were obtained. 

Hue (h*), chroma (C*) and total colour differences (ΔΕ*) in control and coated samples were estimated by the Equations (2), (3) and (4), respectively.

h* = arctg(b*/a*)(2)

C* = (a*^2^ + b*^2^)^1/2^ (3)

ΔΕ* = ((Δa*)^2^ + (Δb*)^2^ + (ΔL*)^2^)^1/2^ (4)

### 2.6. Texture Profile Analysis

Meat samples (n = 6) were subjected to texture profile analysis (TPA) using a TA-TX2 texture analyser (Stable Micro Systems Ltd., Reading, UK). Meat cubes were placed between a stainless-steel plate and probe (50 mm in diameter) and were compressed to 75% of their original height in a double cycle at a rate of 2 mm/s. The texture profile parameters, including hardness, elasticity, adhesiveness, cohesiveness, and chewiness, were calculated from the obtained force-deformation curves.

### 2.7. Lipid Oxidation

Lipid oxidation of uncoated meat samples (control), coated with gelatin, and coated with TBPH-enriched gelatin during storage (3, 6, 8, and 13 days) was evaluated, in duplicate, by the thiobarbituric acid reactive substances (TBARS) method [22]. Briefly, 5 g of the sample was mixed with 10 mg of BHT and 20 mL of 5% TCA, homogenised and centrifuged at 12,000 rpm and 4 °C for 10 min. Then, the supernatant was filtered through a 0.2 μm cellulose membrane filter. The TBARS assay was performed by mixing 4 mL of sample and 4 mL of 20 mM TBA solution, and heating at 100 °C for 1 h. After cooling, the absorbance was measured at 532 nm. BHT was used as positive control and TMP (0.2–12.5 μM) as standard. The results were expressed as mg malonaldehyde (MDA) per kg of the sample.

### 2.8. Statistical Analysis

Statistical analysis, including one-way analysis of variance (ANOVA) and Fisher’s Least Significant Difference (LSD) tests, were performed using Statgraphics Centurion XVII software (Statgraphics Technologies, Inc., The Plains, VA, USA). Results were expressed as the mean of replicates ± standard deviations, and differences were considered significant at *p* < 0.05. 

## 3. Results and Discussion

### 3.1. TBPH Characterisation

#### 3.1.1. Optimisation of Hydrolysis Time in the Preparation of TBPH

**Figure 1:**
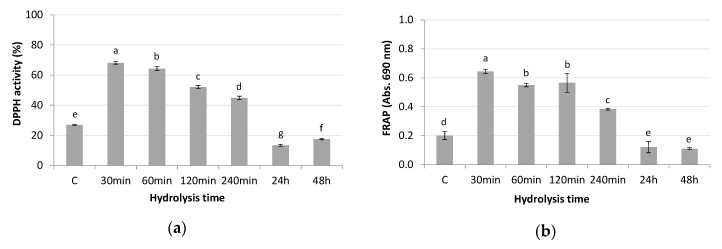
Antioxidant activity determined by (**a**) DPPH radical scavenging activity and (**b**) ferric reducing antioxidant power (FRAP) of tomato by-products hydrolysates obtained at different Alcalase hydrolysis times. a–g: different letters indicate significant differences between hydrolysis times (*p* < 0.05).

Hydrolysis conditions (temperature, pH, pressure, reaction time), as well as the enzyme-substrate ratio and the degree of hydrolysis, are known to influence the specific chemical properties of the resulting peptides [23]. In order to achieve the highest antioxidant activity in the TBPH, different times of hydrolysis by Alcalase enzyme were assayed and the antioxidant activity of the hydrolysates (10 mg/mL) was measured by DPPH radical scavenging activity and FRAP (Figure 1). 

Results obtained from both methods showed the highest activity at 30 min of hydrolysis, with values of 68.08 ± 0.84% DPPH activity and absorbance of 0.64 ± 0.01 at 690 nm using FRAP. Meanwhile, the antioxidant activity decreased when increased times of hydrolysis were used, probably due to excessive protein hydrolysis that could reduce the ability of peptides to act as antioxidants [24]. In fact, enzymatic breakdown of proteins involves the release of amino acids and the generation of peptides with specific characteristics such as size, structure, amino acid composition, and hydrophobicity, which determine their antioxidant activity [25,26]. The obtained DPPH scavenging activity of TBPH (68.08%) was slightly higher than that of tomato seed proteins hydrolysate (62.99%) obtained by Alcalase hydrolysis for 138.62 min [14], as well as than that obtained in tomato seed meals (61.4%) fermented with *Bacillus subtilis* for 12 h [12]. So, the use of tomato skins together with seeds would provide higher antioxidant activity in hydrolysates obtained at shorter hydrolysis times.

#### 3.1.2. Effect of Cooking on Antioxidant Activity of TBPH

**Figure 2:**
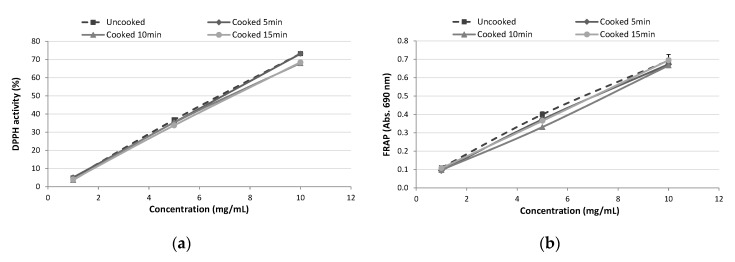
Antioxidant activity determined by (**a**) DPPH radical scavenging activity and (**b**) ferric reducing antioxidant power (FRAP) of tomato by-products hydrolysates before and after cooking at 70 °C (5, 10, and 15 min).

Heat treatment is a common process in food manufacturing that can influence the functional properties of protein hydrolysates. TBPH obtained after 30 min of Alcalase hydrolysis was subjected to cooking at 70 °C for different times (5, 10, and 15 min) and the antioxidant activity was evaluated in order to optimise the conditions to achieve the highest activity. Figure 2 shows that non-significant differences (*p* < 0.05) were found between the control (uncooked) and cooked samples at different times for each assayed concentration in neither DPPH nor FRAP methods. High temperatures can affect the secondary structure of peptides and thus their bioactivity [27]; however, TBPH-derived antioxidant peptides presented good stability against heating. A cooking time of 5 min was chosen for further analyses evaluating the effect of cooking on antioxidant activity of coated meats.

#### 3.1.3. Peptidomic Characterisation

##### Antioxidant Fractionation by SEC and RP-HPLC

**Figure 3:**
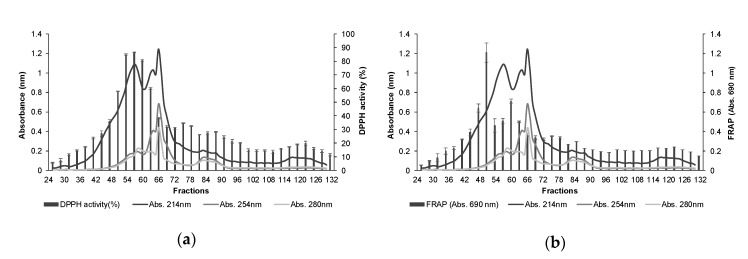
Size-exclusion chromatography profile of tomato by-products hydrolysate and antioxidant activity determined by (**a**) DPPH radical scavenging activity and (**b**) ferric reducing antioxidant power (FRAP) of each of the three pooled fractions.

The TBPH was fractionated by molecular weight using SEC, and the absorbance of each collected fraction was measured at 214, 254, and 280 nm. Factions from 26 to 130 (corresponding to elution volumes from 130 to 650 mL) were selected and then grouped into three for evaluating antioxidant activities (Figure 3). The obtained results showed that fractions 53–55, 56–58, and 59–61, renamed as f10, f11, and f12, respectively, were those showing the highest DPPH activity, with values higher than 80% (Figure 3b). On the other hand, fractions 50–52 (f9) and 59–61 (f12) showed the highest absorbance at 690 nm, and thus the highest FRAP (Figure 3b). Considering these results, fractions f9, f10, f11, and f12 were pooled together and analysed by RP-HPLC.

**Figure 4:**
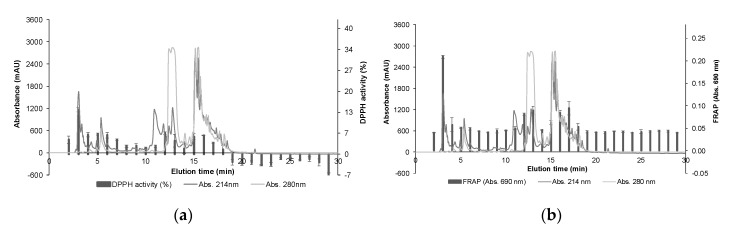
RP-HPLC profile of the selected pooled fractions (f9–f12) of tomato by-products hydrolysate obtained from size-exclusion chromatography, and antioxidant activity determined by (**a**) DPPH radical scavenging activity and (**b**) ferric reducing antioxidant power (FRAP) of the collected fractions.

A total of 30 fractions were obtained from RP-HPLC, which separates peptides based on their polarity. The peptide profile at 214 and 280 nm, as well as the antioxidant activity of the collected fractions, are presented in Figure 4. The fraction eluted at 3 min showed the maximum antioxidant activity in both methods, with 14.15 ± 0.68% DPPH activity and an absorbance value of 0.21 ± 0.01 in FRAP. Additionally, fractions eluted at 12–13 and 16–17 min presented DPPH activity between 3.6–6.7% and FRAP absorbance values higher than 0.08 at 690 nm. The peptide profile obtained at 280 nm suggests the presence of aromatic amino acids in peptide sequences contained in the most active fractions (Figure 4). Previous studies have reported that small peptide size and high level of hydrophobic and aromatic amino acids in their sequences contribute to a strong radical scavenging activity and metal chelation, and therefore would be associated with enhanced antioxidant activity [13,28,29]. Fraction eluted at 3 min, as well as the mixtures of fractions eluted at 12–13 min and 16–17 min, were analysed by MS/MS for peptide identification and further analysed *in silico*.

##### Peptide Identification and *In Silico* Analysis

The identification of the peptides from TBPH was performed by nLC-MS/MS. The complete list containing the identified peptide sequences as well as their protein of origin, mass/charge (m/z), observed and calculated molecular masses, and charge is shown in Table S1 (Supplementary Material). Briefly, a total of 30, 42, and 80 peptides were identified in the RP-HPLC fractions eluted at 3 min, 12–13 min, and 16–17 min, respectively. The identified peptides presented 7–20 amino acids in length and molecular masses between 500 and 2200 Da. These results agree with previous reports that describe most bioactive peptides, including antioxidant peptides, to be between 2 and 20 amino acid residues and molecular masses ranged between 400–3000 Da [30].

Bioinformatic approaches are both cost- and time-effective alternatives to empirical methods. *In silico* analysis of the identified peptides was carried out using several tools and databases in order to evaluate their potential bioactivity, toxicity, allergenicity, and physicochemical properties. The search in the BIOPEP database indicated that neither of the identified peptides was already reported as bioactive, so Peptide Ranker was used to predict their potential to be so. Table 1 presents the list of the 45 identified peptides showing a Peptide Ranker score higher than 0.5, and those sequences found in BIOPEP to share active domains with previously reported antioxidant peptides were highlighted. As an example, the tripeptide GPP contained in peptides DPQYPPGPPAF and NPGPPGT of fractions 16–17 have previously been described to exert an important scavenging activity on DPPH radicals (EC_50_ = 1.93 mg/mL), hydroxyl radicals (EC_50_ = 2.36 mg/mL), and ABTS radicals (EC_50_ = 2.47 mg/mL) [31]. 

The amino acid composition and sequence of the peptides greatly influence the antioxidant activity of peptides. So, hydrophobic amino acids such as proline (P), alanine (A), leucine (L), valine (V), glycine (G), and methionine (M), the aromatic amino acids tyrosine (Y), tryptophan (W), and phenylalanine (F), as well as cysteine (C) and histidine (H) have been reported to be involved in several mechanisms such as free radical scavenging, reduction reactions, metal chelation, and inhibition of lipid peroxidation [25,29]. Additionally, the position of amino acids in the peptide chain provides certain secondary structure and spatial conformation in the peptides resulting in specific physicochemical properties that are also determinants of their antioxidative properties [32,33,34]. On the other hand, 24 of the 45 peptides listed in Table 1 were predicted to be neither toxic nor allergens according to ToxinPred and AllerTop tools. This classification would be based on the amino acid composition and physicochemical properties of the studied peptides [17,18].

### 3.2. Coating of Fresh Pork Loin with Gelatin Enriched with Tomato By-Products Hydrolysate

#### 3.2.1. Physicochemical Characteristics

The effects of gelatin coating enriched with TBPH on meat quality during cold storage (3, 6, 8, and 13 days) were evaluated by the measurement of several physicochemical parameters (Table 2). 

Weight loss tends to increase during storage and it can influence the texture and colour properties of the meat products. In this regard, previous studies have reported the action of gelatin coating as barrier to water, reducing exudates that result in a purge of meat samples [5,6]. In the present study, the weight loss of meat coated with TBPH-enriched gelatin was higher than uncoated samples at 3, 6, and 8 days (Table 2), probably due to the fact that the gelatin removed from the meat would have absorbed surface water from the meat sample. Gelatin has a mixture of single- and double-unfolded chains of hydrophilic character that confer it a high water holding capacity [4]. In fact, the diffusion of moisture through gelatin films would be inversely proportional to the concentration of gelatin in the aqueous solution [35]. At the end of storage, uncoated samples showed a marked weight loss, leading to non-significant differences (*p* < 0.05) between control and coated samples (Table 2).

The pH measurement is important to determine potential microbial growth that could cause meat deterioration. Non-significant differences (*p* < 0.05) in pH values were found in control and coated samples during the first 8 days of cold storage, with values ranging from 5.44 to 5.83 (Table 2). On day 13, the pH was maintained in coated samples but increased up to 7.12 in uncoated samples, probably due to meat spoilage and formation of amines and NH_3_ from amino acid metabolism [36].

The water activity (a_w_) refers to the amount of free water available for bacterial growth and thus can be measured to control the spoilage of stored meats. Results presented in Table 2 revealed a constant a_w_ evolution throughout the 13 days of storage for both uncoated and coated samples, with values between 0.98 and 0.99. So, no significant differences (*p* < 0.05) were found between samples despite the loss of surface water during the first 8 days of cold storage observed from the weight loss data.

Colour is a crucial parameter in food quality control as it determines consumers’ acceptability. Colour deterioration results from oxidation of the meat pigment myoglobin and is affected by many intrinsic and extrinsic factors [1]. Table 2 shows colour attributes (L*, C* and h*) of the studied meat samples during cold storage. Values of lightness (L*) slightly increased during storage and were significantly higher (*p* < 0.05) in meat samples coated with the enriched gelatin than control samples. The increase in lightness over time would be due to changes in meat structure, mainly protein denaturation [37]. Values of croma (C*) of uncoated meat slightly increased during storage time, whereas they remained invariable in the coated samples. Values of hue (h*) of both uncoated and coated meat slightly increased during storage time. Total colour differences (ΔΕ*) increased for control samples as storage time increased, and significant differences (*p* < 0.05) were observed between samples at all the assayed times, with higher colour differences in coated meat samples (Table 2). These results evidence no effect of gelatin coating enriched with TBPH on improving meat colour preservation. Previous studies have evaluated the effects of gelatin-coated beef meat [7] and pork loin [5], reporting lower total colour changes in coated samples in comparison to controls. Conversely, Antoniewski et al. [5] reported that gelatin did not protect pork meat against colour degradation, as coated samples had the same L* and a* values as control samples. The effectiveness of gelatin coating in reducing meat colour deterioration varies between different breeds, species, and muscle types, mainly depending on myoglobin levels. Also, the pH and enzyme activity during postmortem aging, as well as other extrinsic factors such as light, temperature, relative humidity, microbial load, and processing, could lead to colour changes [1].

Texture is also an important sensory parameter for consumer acceptability. Table 2 summarises the values of different parameters (hardness, elasticity, adhesiveness, cohesiveness, and chewiness) obtained for uncoated and coated samples. Results in uncoated samples showed that hardness increased during storage, probably due to aggregation and water loss induced by denaturation of myofibrillar proteins [38]. Non-significant differences (*p* < 0.05) between samples were observed, except for day 13, in which the hardness of the coated sample was higher than the control. In this regard, interactions between coating solutions and tissues could cause changes in texture parameters [39]. Values of elasticity, cohesiveness, and chewiness did not show significant differences (*p* < 0.05) between samples, and only slight variations throughout the cold storage were observed within the same sample. Nevertheless, adhesiveness increased at the end of storage, with a greater slope for control, but a significant reduction (*p* < 0.05) in coated samples in comparison to control was observed at 3 and 6 storage times. These results suggest that gelatin coating would not modify the texture properties of meat during storage, except in the reduction of adhesiveness increase during the first days. The increase in meat adhesiveness leads to unappealing sticky product effects that are undesirable for consumers’ acceptance [40].

#### 3.2.2. Effect of Coating and Enriched Coating on Meat Lipid Oxidation

**Figure 5:**
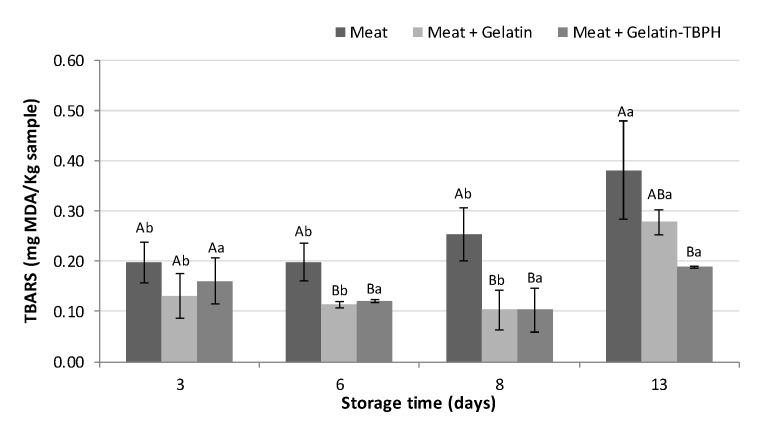
Lipid oxidation of uncoated and coated meat samples during storage (3, 6, 8, and 13 days). A capital letter indicates significant differences between samples within the same day of storage, whereas a lower case letter indicates significant differences between days of storage within the same sample (*p* < 0.05).

Lipid oxidation is an important influential factor limiting the shelf-life of meat. For that, lipid oxidation in terms of TBARS was measured during the cold storage of meat samples uncoated, coated with gelatin, and coated with the enriched gelatin (Figure 5). Results showed an increase in lipid oxidation during the storage period for uncoated and gelatin-coated samples, whereas TBARS values were maintained in the meat coated with TBPH-enriched gelatin. When comparing between samples, controls presented higher TBARS values than coated samples at 6 and 8 days of storage, but non-significant differences (*p* < 0.05) were found between the two coated meats. However, at the end of storage (day 13), control samples reached a maximum value of 0.38 mg MDA/Kg, whereas meat samples coated with gelatin and with enriched gelatin presented values of 0.28 and 0.19 mg MDA/Kg, respectively (Figure 5). 

These results suggest the effectiveness of gelatin coating for preventing lipid oxidation, which can be enhanced with the addition of TBPH extracts with antioxidant activity. Similar results were obtained in gelatin-coated pork meat [6] and beef meats coated with gelatin enriched with henna extracts during chilling storage [7]. The good oxygen barrier properties of gelatin would be given by hydrogen bonds present in gelatin [5] and by the strong polymer-meat interaction created by the presence of ionic compounds [41]. Additionally, gelatin contains peptides and amino acids that may act as electron donors and exert antioxidant activity, although this activity would be exerted to a greater extent when gelatin is hydrolysed. In this case, the generated peptides may expose hidden amino acid residues and side chains with antioxidant properties, as well as the release of glycine and proline residues which would play an important role in radical scavenging [42]. Moreover, the antioxidant activity of coatings is greatly influenced by water availability. In high moisture products such as meat, coating becomes plasticised due to film-water molecules interaction, increasing the permeability of oxygen, and thus the specific activity of added antioxidant compounds could become more relevant [43]. In this regard, the antioxidant activity of TBPH could counteract lipid oxidation by protecting target lipids from oxidation initiators or by delaying the propagation phase, which could be achieved through different mechanisms such as free radical scavenging, inactivation of peroxides and other reactive oxygen species (ROS), chelation of metals, and quenching of secondary lipid oxidation products [44].

#### 3.2.3. Effect of Cooking on Antioxidant Activity of Coated Meats

**Figure 6:**
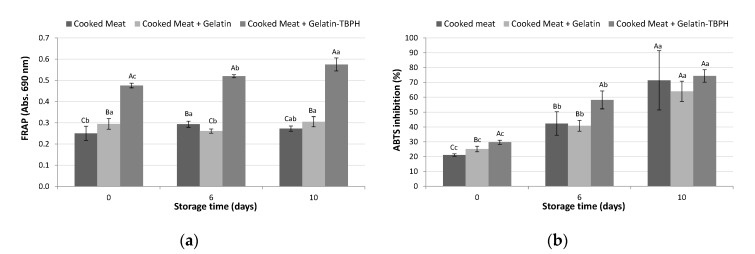
Antioxidant activity determined by (**a**) ferric reducing antioxidant power (FRAP) and (**b**) ABTS radical scavenging capacity of uncoated and coated meat samples during storage (0, 6, and 10 days) after cooking at 70 °C for 5 min. A capital letter indicates significant differences between samples within the same day of storage, whereas a lower case letter indicates significant differences between days of storage within the same sample (*p* < 0.05).

The antioxidant activity of meat samples including uncoated, coated with gelatin and coated with TBPH-enriched gelatin was evaluated by FRAP and ABTS methods after subjecting samples to cooking (70 °C, 5 min) at different storage times (0, 6, and 10 days) (Figure 6). The results of the present study did not show a substantial increase in the antioxidant activity of samples coated with gelatin in comparison to uncoated samples in neither FRAP nor ABTS assays (Figure 6). Nevertheless, meat samples coated with TBPH-enriched gelatin showed the highest antioxidant activity measured by FRAP at all the assayed storage times, showing a marked difference with regard to the other samples due to the antioxidant activity given by the hydrolysate that is maintained after cooking (Figure 6a). In the ABTS assay, increased antioxidant activity of samples coated with the enriched gelatin was observed at 0 and 6 days, but similar values close to 70% inhibition were obtained for all the samples at 13 days of storage. These results suggest the positive role of gelatin films as carriers of antioxidant compounds for improving the antioxidant activity of cooked meats. However, when comparing the FRAP activity between the TBPH cooked for 5 min (Figure 2b) and the cooked meat sample containing the TBPH-enriched gelatin (Figure 6a), a reduction in absorbance values from 0.67 to 0.57 was observed. This could be attributed to interactions between the TBPH-derived peptides and gelatin film matrix formed via hydrogen bonding [42].

On the other hand, the activity of cooked TBPH-enriched gelatin-coated samples significantly (*p* < 0.05) increased over storage time, ranging from absorbance values from 0.48 at 0 day to 0.57 at 10 days in FRAP (Figure 6a). In the ABTS assay, inhibition values increased up to 40% from day 0 to 10 in all the assayed samples (Figure 6b). Cooking and storage are important in the development of denaturation and oxidation processes that can affect the structural properties and physicochemical states of proteins and peptides [45]. So, the unfolding of proteins can increase their radical scavenging ability by increasing the exposure of antioxidant amino acids that would normally be located in the core of the protein structure [46]. Furthermore, Maillard reaction products, formed via amino-carbonyl compound interactions during cooking and storage, have been reported to exhibit strong antioxidant activity through several mechanisms, including scavenging of ROS, hydroperoxide decomposition, and metal chelation, thus retarding the formation of oxidation products [47].

## 4. Conclusions

TBPH obtained from Alcalase hydrolysis showed antioxidant activity with good stability against cooking. The application of TBPH to enrich gelatin coating led to some changes in meat appearance, such as an increased weight loss and small colour differences, but maintained the pH and water activity that control microbial spoilage and the texture of meat. Additionally, gelatin coatings decreased lipid oxidation of meats during storage, and enriched-coated meats after cooking presented high antioxidant activity. These results suggest a positive role of gelatin coating enriched with antioxidant TBPH in extending the shelf-life of meat during storage. In addition, the use of by-products for obtaining coatings and functional compounds would be a sustainable strategy for the food packaging industry. Further studies are needed in order to identify those TBPH-derived peptides responsible for the observed antioxidant activities as well as to evaluate potential physiological effects in humans after ingestion of coated meats.

## Figures and Tables

**Table 1:** *In silico* prediction of the physicochemical properties, toxicity, and allergenicity of the identified peptides with Peptide Ranker score > 0.5.

RP-HPLC Fractions	Peptide Sequence	Peptide Ranker Score	Hydrophobicity	Hydrophilicity	Steric Hindrance	Amphipathicity	Toxicity Prediction	Allergenicity Prediction
3	GGPAAGCCCRDCCVE	0.95	−0.10	0.10	0.62	0.25	Toxic	Allergen
	GGFGGMC	0.95	0.22	−0.69	0.64	0.00	Non-toxic	Non-allergen
	LLIVILFLTIC	0.70	0.48	−1.64	0.62	0.00	Non-toxic	Non-allergen
	PAAQPGC	0.68	−0.02	−0.26	0.53	0.18	Non-toxic	Non-allergen
	EFTCPNC	0.66	−0.12	−0.24	0.61	0.18	Non-toxic	Allergen
	EQAPACAMG	0.66	−0.02	−0.07	0.60	0.28	Non-toxic	Non-allergen
	GCNGEPC	0.65	−0.13	0.17	0.63	0.18	Non-toxic	Allergen
	C**CQC**SYA	0.55	−0.08	−0.76	0.61	0.18	Toxic	Non-allergen
12-13	SCPCCGT	0.89	−0.03	−0.44	0.57	0.00	Toxic	Allergen
	LPSECG**FC**	0.87	0.05	−0.38	0.59	0.16	Non-toxic	Allergen
	PGGAGPC	0.81	0.09	−0.21	0.56	0.00	Non-toxic	Non-allergen
	CLATC**FC**PN	0.78	0.07	−0.89	0.58	0.00	Non-toxic	Allergen
	VPSGCFEGGAGNC	0.77	0.04	−0.23	0.63	0.10	Non-toxic	Non-allergen
	SEYCCCSC	0.77	−0.12	−0.34	0.61	0.16	Toxic	Allergen
	QCGEGMC	0.69	−0.09	−0.01	0.68	0.36	Non-toxic	Allergen
	CSQGEGSYEGPLG	0.66	−0.10	0.13	0.62	0.29	Non-toxic	Non-allergen
	SGADPAC	0.64	−0.05	0.19	0.57	0.00	Non-toxic	Allergen
	SGGGACSDTGACTPAR	0.58	−0.12	0.14	0.59	0.15	Non-toxic	Allergen
	GRGGGAC	0.54	−0.12	0.21	0.65	0.35	Non-toxic	Non-allergen
	AN**GAA**GC	0.54	0.07	−0.33	0.61	0.00	Non-toxic	Allergen
16-17	DP**QYP**P**GPP**AF	0.88	−0.07	−0.19	0.53	0.11	Non-toxic	Non-allergen
	CWQDPSMDMH	0.84	−0.19	−0.10	0.58	0.27	Non-toxic	Non-allergen
	GKCECGQCTCFP	0.83	−0.13	−0.06	0.62	0.52	Toxic	Allergen
	IHGGGWC	0.80	0.17	−0.96	0.55	0.21	Non-toxic	Non-allergen
	AIILFFVCILV	0.78	0.53	−1.68	0.65	0.00	Non-toxic	Non-allergen
	NP**GPP**GT	0.71	−0.10	−0.03	0.53	0.00	Non-toxic	Non-allergen
	GPSPQAC	0.70	−0.09	−0.14	0.54	0.18	Non-toxic	Non-allergen
	GSPGEPM	0.69	−0.06	0.29	0.58	0.18	Non-toxic	Allergen
	SLA**LY**LP	0.68	0.22	−1.13	0.53	0.00	Non-toxic	Allergen
	LPGGARC	0.67	−0.10	−0.04	0.58	0.35	Non-toxic	Non-allergen
	SPGRGGG	0.64	−0.21	0.47	0.61	0.35	Non-toxic	Non-allergen
	DCSDGSDEKNCDCG	0.64	−0.38	1.13	0.67	0.35	Toxic	Non-allergen
	G**PEL**PPVP	0.63	0.04	−0.04	0.50	0.16	Non-toxic	Allergen
	MGDTGPCG	0.63	−0.02	0.04	0.64	0.00	Non-toxic	Non-allergen
	GGGSPPA	0.61	0.05	−0.03	0.54	0.00	Non-toxic	Non-allergen
	LCSWPGGQSSGVPG	0.60	0.04	−0.47	0.58	0.09	Non-toxic	Non-allergen
	GCCI**LY**S	0.58	0.18	−1.09	0.63	0.00	Toxic	Allergen
	GGGGGHP	0.56	0.05	−0.07	0.54	0.21	Non-toxic	Non-allergen
	NPSLSGC	0.56	−0.07	−0.29	0.57	0.00	Non-toxic	Allergen
	SGQGTPFSYSVPG	0.53	−0.01	−0.43	0.59	0.10	Non-toxic	Non-allergen
	YGGGGGR	0.53	−0.13	0.10	0.68	0.35	Non-toxic	Non-allergen
	APKRQSPLP	0.53	−0.36	0.47	0.52	0.82	Non-toxic	Non-allergen
	ICCGIG**AY**	0.51	0.27	−1.05	0.65	0.00	Toxic	Non-allergen
	AGF**GAA**N	0.51	0.15	−0.54	0.63	0.00	Non-toxic	Allergen
	PSEPTTFGPT	0.51	−0.09	−0.04	0.53	0.13	Non-toxic	Non-allergen

Bold letters indicate active domains previously reported as antioxidant sequences according to BIOPEP database.

**Table 2:** Changes in physicochemical parameters of uncoated meat and meat coated with gelatin enriched with tomato by-products hydrolysate (TBPH) during cold storage.

Parameter		Sample	Storage Time (days)				
			0	3	6	8	13
Weigth loss (%)		Meat		2.09 ± 0.52 ^Bb^	2.98 ± 1.29 ^Bb^	3.18 ± 0.59 ^Bb^	8.08 ± 3.35 ^Aa^
		Meat + Gelatin-TBPH		4.39 ± 0.41 ^Ab^	7.19 ± 1.08 ^Aa^	6.22 ± 0.80 ^Aab^	7.73 ± 3.15 ^Aa^
pH		Meat	5.60 ± 0.02 ^Ab^	5.46 ± 0.11 ^Ab^	5.50 ± 0.23 ^Ab^	5.83 ± 0.36 ^Ab^	7.12 ± 0.28 ^Aa^
		Meat + Gelatin-TBPH	5.60 ± 0.02 ^Aa^	5.57 ± 0.13 ^Aa^	5.63 ± 0.50 ^Aa^	5.44 ± 0.04 ^Aa^	5.88 ± 0.21 ^Ba^
a_w_		Meat	0.98 ± 0.00 ^Aa^	0.98 ± 0.00 ^Aa^	0.98 ± 0.00 ^Aa^	0.98 ± 0.00 ^Aa^	0.98 ± 0.00 ^Aa^
		Meat + Gelatin-TBPH	0.98 ± 0.00 ^Aa^	0.99 ± 0.00 ^Aa^	0.99 ± 0.00 ^A^	0.98 ± 0.00 ^Aa^	0.99 ± 0.00 ^Aa^
Colour	L*	Meat	43.37 ± 2.40 ^Ac^	44.13 ± 1.70 ^Bbc^	42.68 ± 1.60 ^Bc^	47.11 ± 2.37 ^Ba^	45.44 ± 3.91 ^Bb^
		Meat + Gelatin-TBPH	43.37 ± 2.40 ^Ac^	50.42 ± 1.44 ^Ab^	52.00 ± 1.41 ^Aa^	51.56 ± 2.15 ^Aa^	51.73 ± 2.39 ^Aa^
	C*	Meat	7.21 ± 0.85 ^Abc^	6.92 ± 1.01 ^Ac^	6.97 ± 0.87 ^Ac^	7.55 ± 0.81 ^Ab^	9.47 ± 1.08 ^Aa^
		Meat + Gelatin-TBPH	7.21 ± 0.85 ^Aa^	7.28 ± 0.73 ^Aa^	7.09 ± 0.91 ^Aa^	7.38 ± 1.00 ^Aa^	7.53 ± 0.74 ^Ba^
	h*	Meat	86.69 ± 6.42 ^Ab^	83.11 ± 4.82 ^Ac^	79.93 ± 3.37 ^Bd^	83.34 ± 5.73 ^Bc^	91.54 ± 6.75 ^Aa^
		Meat + Gelatin-TBPH	86.69 ± 6.42 ^Ab^	80.30 ± 7.77 ^Ac^	86.38 ± 5.81 ^Ab^	88.87 ± 7.44 ^Aab^	91.10 ± 6.57 ^Aa^
	ΔΕ*	Meat		3.50 ± 1.62 ^Bbc^	2.77 ± 1.46 ^Bc^	4.51 ± 2.59 ^Bb^	5.91 ± 2.89 ^Ba^
		Meat + Gelatin-TBPH		7.26 ± 2.30 ^Aa^	8.80 ± 2.47 ^Aa^	8.41 ± 3.44 ^Aa^	8.50 ± 2.71 ^Aa^
TPA	Hardness (N)	Meat	150.50 ± 34.48 ^Ab^	158.30 ± 24.90 ^Aab^	164.07 ± 39.23 ^Aab^	196.72 ± 45.67 ^Aa^	156.46 ± 19.04 ^Bab^
		Meat + Gelatin-TBPH	150.50 ± 34.48 ^Ab^	145.02 ± 37.70 ^Ab^	192.07 ± 22.67 ^Aab^	154.38 ± 60.86 ^Ab^	210.48 ± 31.35 ^Aa^
	Elasticity	Meat	0.35 ± 0.04 ^Aab^	0.34 ± 0.03 ^Aab^	0.32 ± 0.05 ^Aab^	0.29 ± 0.03 ^Ab^	0.39 ± 0.09 ^Aa^
		Meat + Gelatin-TBPH	0.35 ± 0.04 ^Aa^	0.31 ± 0.03 ^Aab^	0.34 ± 0.05 ^Aa^	0.29 ± 0.02 ^Ab^	0.32 ± 0.04 ^Aab^
	Adhesiveness (N·s)	Meat	−0.38 ± 0.14 ^Ab^	−0.51 ± 0.11 ^Ab^	−0.74 ± 0.37 ^Ab^	−0.60 ± 0.24 ^Ab^	−2.00 ± 1.48 ^Aa^
		Meat + Gelatin-TBPH	−0.38 ± 0.14 ^Ab^	−0.22 ± 0.11 ^Bb^	−0.31 ± 0.13 ^Bb^	−0.39 ± 0.28 ^Ab^	−0.89 ± 0.38 ^Aa^
	Cohesiveness	Meat	0.33 ± 0.07 ^Aab^	0.31 ± 0.05 ^Ab^	0.40 ± 0.07 ^Aa^	0.39 ± 0.06 ^Aab^	0.41 ± 0.07 ^Aa^
		Meat + Gelatin-TBPH	0.33 ± 0.07 ^Ac^	0.31 ± 0.05 ^Ac^	0.40 ± 0.05 ^Ab^	0.33 ± 0.05 ^Ac^	0.45 ± 0.03 ^Aa^
	Chewiness (N)	Meat	17.50 ± 7.85 ^Aa^	17.05 ± 6.02 ^Aa^	22.08 ± 10.91 ^Aa^	23.59 ± 10.35 ^Aa^	25.02 ± 9.06 ^Aa^
		Meat + Gelatin-TBPH	17.50 ± 7.85 ^Ab^	14.49 ± 6.02 ^Ab^	26.60 ± 9.95 ^Aa^	15.38 ± 8.00 ^Ab^	30.07 ± 5.43 ^Aa^

Results were expressed as the mean of 6 replicates ± standard deviations. Capital letter indicates significant differences between samples within the same day of storage, whereas lower case letter indicates significant differences between days of storage within the same sample (*p* < 0.05).

## Supplementary Materials

The following are available online at https://www.mdpi.com/2073-4360/12/5/1032/s1, Table S1: Peptide sequences identified by nLC-MS/MS in RP-HPLC fractions 3, 12-13, and 16-17.
