# Supplementary material for: Effect of Gelatin Coating Enriched with Antioxidant Tomato By-Products on the Quality of Pork Meat
Source: Polymers (Basel). 2020 May 2;12(5):1032. doi: 10.3390/polym12051032 (PMC7284921; doi:10.3390/polym12051032)
Supplement: Supplementary file 1 [file polymers-12-01032-s001.pdf]

Table S1. Peptide sequences identified by nL-MS/MS in RP-HPLC fractions 3, 12-13, and 16-17.

RP-HPLC fraction	P_o[a]	Peptide sequence	P_f[b]	Protein accession number[c]	Observed (*m/z*)	Observed MW (Da)[d]	Charge (+)	Calculated MW (Da)[e]
3	Q	GDGPEGS	G	UIMC1_HUMAN	618.26	617.25	1	617.23
	A	TIDALGN	E	KCNS1_OTOGA	703.39	702.38	1	702.35
	Q	CCQCSYA	S	CTCFL_HUMAN	389.12	776.23	2	776.23
	T	CSNPSIQATMNN	N	CRTC3_XENLA	640.25	1278.48	2	1278.53
	P	CVCTEADF	D	SORC1_HUMAN	444.19	886.36	2	886.32
	K	GAKPVVV	L	NIPBL_HUMAN	335.22	668.43	2	668.42
	C	GNMCGQQ	K	RON_HUMAN	369.12	736.23	2	736.26
	V	CIGKTCDTSCCQ	-	MTA_CHAAC	631.21	1260.40	2	1260.46
	V	KRQARKGSSKKVV	R	RNF8_RAT	736.49	1470.97	2	1470.91
	S	FSSDSPQDD	T	BICRA_HUMAN	499.18	996.34	2	996.37
	S	ESSMSDFSED	E	UBE2H_BOVIN	567.18	1132.35	2	1132.39
	L	GCNGEPC	F	ASB2_BOVIN	340.12	678.22	2	678.21
	S	CTSCGSN	K	VM3_BUNFA	336.11	670.21	2	670.21
	G	GNGGDVC	L	GTPBA_XENLA	311.13	620.24	2	620.22
	G	YGNQNMQGDG	F	TET2_HUMAN	542.19	1082.36	2	1082.41
	Q	TGNGCSV	A	LSHR_PIG	637.24	636.23	1	636.25
	K	APKKKLP	Q	DMAP1_HUMAN	391.28	780.56	2	780.52
	A	GGPAAGCCCRDCCVE	P	KCC1A_HUMAN	722.25	1442.49	2	1442.49
	P	PGAHGSC	H	KHNYN_HUMAN	628.22	627.21	1	627.24
	E	PAAGADC	K	BHLH9_HUMAN	604.23	603.22	1	603.23
	A	EQAPACAMG	P	ATS16_HUMAN	439.16	876.31	2	876.35
	R	ESHAASN	D	HMHA1_MOUSE	358.15	714.29	2	714.29
	F	PAAGKKVL	S	CCNA1_MOUSE	392.27	782.52	2	782.50
	E	LLIVILFLTIC	F	SKIT8_MOUSE	252.98	1259.84	5	1259.79
	K	AGVSCTC	R	DEF5_RABIT	640.24	639.24	1	639.24
	P	PAAQPGC	H	GCM1_MOUSE	322.15	642.28	2	642.28
	A	GGFGGMC	L	MCAT_MOUSE	628.25	627.24	1	627.21
	L	LLLLPPELR	D	MLEC_RAT	532.36	1062.70	2	1062.68
	A	EFTCPNC	T	RYDEN_DANRE	407.13	812.25	2	812.28
	P	SPGGDAV	E	GANP_HUMAN	602.25	601.24	1	601.27
12-13	H	MSEHTKC	P	NUFP1_HUMAN	418.16	834.31	2	834.34
	A	VGGTPSDC	R	TYRO3_HUMAN	368.13	734.25	2	734.29
	T	PEAARLHSF	P	ASB14_MOUSE	514.25	1026.48	2	1026.52
	C	VEEAGFAQC	V	AGRIN_MOUSE	477.19	952.36	2	952.40
	L	IMAGSGC	E	HOGA1_XENLA	638.24	637.23	1	637.26
	C	AVANGGC	Q	LRP1_MOUSE	296.12	590.23	2	590.25
	L	VCVCSGVC	E	HEAT1_DANRE	385.16	768.31	2	768.30
	G	PECQQQCEC	V	SREC_HUMAN	534.19	1066.37	2	1066.35
	N	LEGSYKCEC	E	LDLR_BOVIN	516.19	1030.36	2	1030.41
	C	GNMCGQQ	K	RON_HUMAN	369.12	736.23	2	736.26
	R	SGADPAC	A	PLPP6_RAT	620.22	619.22	1	619.23
	L	TAILKTNTVKVPKPIKVLDA	P	KT3K_HUMAN	430.69	2148.39	5	2148.32
	E	ADADAYEKC	P	F204A_MOUSE	493.18	984.35	2	984.39
	T	CKDTSTGVHCSC	P	SCUB2_MOUSE	620.74	1239.47	2	1239.47
	N	IIVSIKTGLP	L	PECR_PONAB	208.95	1039.71	5	1039.66
	N	QNSEDTVQD	C	SPZ1_BOVIN	518.20	1034.39	2	1034.42
	A	GQGTDDVGLDSS	T	UN13C_MOUSE	575.72	1149.43	2	1149.48
	N	CLATCFCPN	K	CRVP3_DISTY	486.19	970.37	2	970.37
	L	VHCEDCSC	L	SEML_HUMAN	448.12	894.23	2	894.27
	W	VDAHMNC	E	EXT2_BOVIN	395.16	788.30	2	788.29
	-	MSDDNGSGNNC	T	LST1_MOUSE	557.17	1112.32	2	1112.35
	V	SGGGACSDTGACTPAR	S	HOOK1_HUMAN	705.76	1409.51	2	1409.57
	T	PVDNSTP	V	MUC17_HUMAN	365.16	728.30	2	728.33
	A	SEYCCCSC	T	CAHM4_MOUSE	449.12	896.22	2	896.22
	F	LIILVLFPI	A	CYB_HETAO	208.95	1039.71	5	1039.70
	E	LPSECGFC	L	CYHR1_BOVIN	428.17	854.33	2	854.33
	-	MADSDEWDADNF	E	EI3JB_DANRE	708.27	1414.52	2	1414.50
	P	VPSGCFEGGAGNC	S	KCNC2_MOUSE	599.22	1196.42	2	1196.46
	G	AGGQHAC	A	OPLA_BOVIN	643.25	642.24	1	642.25
	L	ANGAAGC	V	MFRN2_DANRE	282.11	562.21	2	562.22
	S	CEGANQS	D	ZO29_XENLA	708.26	707.25	1	707.25
	Q	LKVLLSGATFLV	T	ZFY21_HUMAN	252.97	1259.84	5	1259.79
	S	GLISTKKRKMV	E	PK1IP_HUMAN	252.97	1259.83	5	1259.77
	V	PGGAGPC	A	CARM1_HUMAN	558.23	557.22	1	557.23
	A	KVPLGGV	Q	CK016_HUMAN	335.22	668.43	2	668.42
	P	GASSPEC	R	LYAG_BOVIN	650.25	649.24	1	649.24
	G	GRGGGAC	S	AHDC1_HUMAN	577.22	576.22	1	576.24
	K	CSQGEGSYEGPLG	P	ROS1_MOUSE	642.24	1282.46	2	1282.51
	V	LVKRHVKL	H	CADHB_XENLA	248.93	991.71	4	991.67
	L	QCGEGMC	C	VAR1_VARVA	364.10	726.19	2	726.21
	K	SCPCCGT	P	RNF14_HUMAN	670.18	669.17	1	669.19
	Q	NCHCHSD	L	CHAD_BOVIN	815.22	814.21	1	814.25

16-17	E	IFKSGLT	T	ANGP2_MOUSE	383.22	764.42	2	764.44
	R	SLALYLP	G	CP090_MOUSE	388.72	775.42	2	775.45
	E	TAASCEDF	Q	WFKN1_RAT	843.29	842.28	1	842.31
	R	DPQYPPGPPAF	P	TITIN_HUMAN	593.28	1184.55	2	1184.55
	R	KVLSGVVMT	T	RED1_MOUSE	467.29	932.56	2	932.54
	G	AKVVVLPEC	F	NIT2_DANRE	479.29	956.56	2	956.54
	C	DFLEDGEERN	C	NGP_MOUSE	612.26	1222.50	2	1222.51
	V	SGTKEGM	V	FAN_MOUSE	709.29	708.28	1	708.31
	G	SGQGTPFSYSVPG	Q	NPTXR_MOUSE	642.29	1282.57	2	1282.58
	Q	EAMDVTTTST	R	EIF3I_BOVIN	528.21	1054.41	2	1054.45
	D	GCCILYS	D	AGRA3_MOUSE	758.31	757.30	1	757.31
	F	VCQATGE	P	PTPRF_BOVIN	707.29	706.28	1	706.30
	M	APKRQSPLP	L	SETLP_HUMAN	497.27	992.53	2	992.58
	Q	VVSTKVG	G	PIGA_MOUSE	345.22	688.42	2	688.41
	P	KTGLLMT	I	MAGBA_CANLF	382.24	762.46	2	762.43
	G	LPGGARC	F	NDUS6_BOVIN	337.17	672.32	2	672.34
	-	MAEAAIDPRCEEQE	E	LSP1_MOUSE	796.32	1590.63	2	1590.67
	L	NPSLSGC	Y	CDON_MOUSE	339.15	676.28	2	676.29
	F	VNVASFC	G	GPX6_RAT	370.19	738.36	2	738.34
	S	SPGRGGG	G	SOCS7_HUMAN	294.16	586.31	2	586.28
	P	GPELPPVP	F	SOCS7_HUMAN	403.23	804.45	2	804.44
	T	PGELVKY	L	LOXE3_MOUSE	403.23	804.45	2	804.44
	Q	SPAAKGG	T	GEMI8_BOVIN	294.16	586.31	2	586.31
	E	DAGGLHVLLTAT	P	INSRR_HUMAN	584.30	1166.58	2	1166.63
	K	SAHCENT	L	OFD1_MOUSE	381.14	760.26	2	760.28
	A	METHNGAED	M	LRRK1_MOUSE	502.19	1002.36	2	1002.37
	N	VNTCPSAS	P	CA174_XENLA	778.31	777.30	1	777.33
	T	LCSWPGGQSSGVPG	L	UFSP1_MOUSE	666.28	1330.55	2	1330.60
	V	GPNSVVC	A	RON_HUMAN	338.15	674.28	2	674.31
	C	GNMCGQQ	K	RON_HUMAN	369.12	736.23	2	736.26
	A	DCEDQGA	P	DBF4B_XENLA	369.12	736.23	2	736.23
	N	NPSGVVC	D	CYB_GENMA	338.15	674.28	2	674.31
	A	CLYYCSKDS	S	NHLC2_CHICK	541.22	1080.42	2	1080.43
	V	DDNEPEC	Q	ADAM7_MOUSE	821.29	820.28	1	820.25
	L	ESDSLTDSES	L	MAGC1_HUMAN	535.21	1068.40	2	1068.41
	W	PSEPTTFGPT	G	OTUD4_MOUSE	517.24	1032.47	2	1032.48
	S	EYTCGTSPAD	K	HXA9_HETFR	522.23	1042.44	2	1042.39
	A	AIMSDTDSDE	D	TAF1_HUMAN	542.19	1082.37	2	1082.41
	E	CWQDPSMDMH	N	N42L2_RAT	625.22	1248.43	2	1248.44
	A	ESDEDEAH	G	EH1L1_HUMAN	466.15	930.28	2	930.32
	E	MGQNEPDQG	G	HCD2_BOVIN	488.18	974.34	2	974.38
	S	GTMSGPD	P	NUBP1_XENTR	664.25	663.24	1	663.25
	H	DTGCIQY	M	TEN4_DANRE	400.16	798.31	2	798.32
	E	SEDEFGPNS	F	TEN4_DANRE	491.18	980.35	2	980.37
	C	ICCGIGAY	P	CHPT1_DANRE	400.16	798.31	2	798.34
	I	MVEEFVQYGPL	D	JAK1_CYPCA	656.32	1310.62	2	1310.62
	P	GSPGEPM	E	STKL1_HUMAN	674.27	673.26	1	673.27
	G	ESGSSSSKTSTHS	K	FIBA_BOVIN	641.26	1280.51	2	1280.55
	L	SMTQSETTSDQSDI	E	CE350_HUMAN	765.29	1528.57	2	1528.62
	V	MTMQHGQ	P	UTP15_DANRE	832.31	831.30	1	831.34
	S	YGGGGGR	Y	RBMX_BOVIN	623.32	622.31	1	622.28
	A	GGGSPPA	T	LRC4B_HUMAN	542.25	541.24	1	541.25
	K	MPSASGH	R	OR5DG_HUMAN	686.27	685.27	1	685.29
	L	LLILTKT	G	CMKMT_MOUSE	401.29	800.56	2	800.54
	E	IISKLLS	E	CMIP_XENLA	387.27	772.53	2	772.51
	G	GPSPQAC	P	EI2BD_RAT	330.15	658.28	2	658.27
	Y	IHGGGWC	F	AAAD_BOVIN	365.14	728.27	2	728.31
	G	GGLVGYG	L	MSGN1_CHICK	622.33	621.32	1	621.31
	S	AGFGAAN	L	TRFE_GADMO	304.15	606.28	2	606.28
	L	AIKAGEA	D	TRFE_GADMO	330.19	658.36	2	658.37
	N	AIKGGVD	D	A2MG_BOVIN	330.19	658.36	2	658.37
	F	AIILFFVCILV	I	S5A4A_MOUSE	250.97	1249.81	5	1249.75
	L	NPGPPGT	P	ZAR1_TAKRU	320.16	638.31	2	638.30
	S	KKAVSLR	T	RPGR1_MOUSE	401.29	800.56	2	800.52
	V	AMELANMTSKQ	M	NU5M_CARAU	612.30	1222.58	2	1222.57
	H	MDSDSSP	S	RNAS1_GORGO	738.23	737.23	1	737.25
	R	GKCECGQCTCFP	P	ITGBL_XENLA	638.22	1274.42	2	1274.46
	E	MGDTGPCG	P	SYAC_HUMAN	369.12	736.23	2	736.25
	N	IIVSIKTGLP	L	PECR_PONAB	208.95	1039.71	5	1039.66
	G	GGGGGHP	H	MAF_CHICK	269.64	537.26	2	537.23
	Q	GKGGHLA	A	SLA_PHIOL	320.18	638.35	2	638.35
	N	FIKVPVV	P	PROC_FELCA	401.28	800.54	2	800.52
	S	IIIIIHLVEVV	P	CALCR_MOUSE	252.97	1259.83	5	1259.82
	A	ASAAGGK	K	ABT1_BOVIN	281.14	560.27	2	560.29
	G	FTGSNCEEECM	N	COQA1_MOUSE	625.21	1248.41	2	1248.41
	S	MHSAGGG	S	FOXF1_HUMAN	616.25	615.24	1	615.24

A	LGGAGGK	G	MTG2_HUMAN	280.17	558.32	2	558.31
Q	ESDPEDDD	V	PININ_BOVIN	461.14	920.26	2	920.29
T	DCSDGSDEKNCDCG	L	ST14_MOUSE	724.23	1446.44	2	1446.43
I	YGAVGSQ	V	MYP0_MOUSE	341.18	680.35	2	680.31

[a]Position of the amino acid residue preceding the peptide sequence.

[b]Position of the amino acid residue following the peptide sequence.

[c]Protein accession number according to UniProt database.

[d]Observed molecular weight (Da) of the identified peptide.

[e]Calculated molecular weight (Da) of the matched peptide.
